# Experimental Evidence on the Impact of Food Advertising on Children's Knowledge about and Preferences for Healthful Food

**DOI:** 10.1155/2013/408582

**Published:** 2013-04-17

**Authors:** Lucia A. Reisch, Wencke Gwozdz, Gianvincenzo Barba, Stefaan De Henauw, Natalia Lascorz, Iris Pigeot

**Affiliations:** ^1^Copenhagen Business School, Porcelaenshaven 18, 2000 Frederiksberg, Denmark; ^2^National Research Council, Institute of Food Sciences, Via Roma, 52 A/C, 83100 Avellino, Italy; ^3^Ghent University, De Pintelaan 185 Blok. A-2, 9000 Ghent, Belgium; ^4^University of Zaragoza, Domingo Miral s/n, 50009 Zaragoza, Spain; ^5^University of Bremen, Achterstraße 30, 28359 Bremen, Germany

## Abstract

To understand the rising prevalence of childhood obesity in affluent societies, it is necessary to take into account the growing obesity infrastructure, which over past decades has developed into an obesogenic environment. This study examines the effects of one of the constituent factors of consumer societies and a potential contributory factor to childhood obesity: commercial food communication targeted to children. Specifically, it investigates the impact of TV advertising on children's food knowledge and food preferences and correlates these findings with their weight status. Evaluations of traditional information- and education-based interventions suggest that they may not sustainably change food patterns. Based on prior consumer research, we propose five hypotheses, which we then test using a subsample from the IDEFICS study, a large-scale pan-European intervention study on childhood obesity. The results indicate that advertising has divergent effects on children's food knowledge and preferences and that food knowledge is unrelated to food preferences. This finding has important implications for both future research and public policy.

## 1. Background and Aim of the Study

In consumer societies, modern diets based on unhealthy fast foods, convenience foods, energy dense snacks, and soft drinks, the abundance and omnipresence of food, and sedentary lifestyles and electronic recreation that minimises physical activity have led to serious weight control problems. A particularly severe trend impacting future health levels are the high, and in most countries still rising, levels of overweight and obesity in infants and children [1]. According to statistics provided by the World Health Organization [2], the Organisation for Economic Co-operation and Development [3], and the International Obesity Task Force (IOTF) (http://www.iaso.org/iotf/obesity/), the problem is increasing and steadily affecting many low- and middle-income countries. Globally, the number of overweight children under the age of five was estimated in 2010 to be over 42 million; close to 35 million of them living in developing countries. About 60% of children who are overweight before puberty will be overweight in early adulthood [4]. 

On an individual level, childhood obesity is strongly associated with risk factors for type 2 diabetes, cardiovascular disease, underachievement in school, and lower self-esteem. On a social level, it jeopardises societies' sustainability through the erosion of social cohesion, equity, and fairness. In the developed world, obesity is closely connected with low socioeconomic status (SES); that is, membership in groups for whom access to and availability and affordability of healthier food choices and physical activity is particularly limited [5]. There is also evidence that cumulative exposure to television food advertising—which is higher in lower SES groups—is linked to adult fast-food consumption [6].

Beyond individual and social problems, rising obesity rates impact healthcare systems and labour markets and also carry environmental costs: modern diets in consumer societies, high in processed foods and animal protein, have a particularly negative ecological footprint—a long neglected fact that has given rise to a debate on “globesity” [7]. Put simply, halting and reversing current childhood obesity trends is not simply an imperative for public health policies but rather is increasingly understood as a broader societal challenge that has become an explicit goal of sustainability strategies worldwide [8]. As a result, addressing obesity among children and adolescents has become a top public health priority—particularly in the USA, which has one of the highest incidences of obesity worldwide [9].

### 1.1. Drivers and Impact of Childhood Obesity

In light of these challenges, researchers and policy makers have been focusing on the key drivers and barriers for healthy diets and healthy lives in childhood. Based on scientific evidence on the importance of the immediate “choice context” of the socialisation environment in which children acquire their food knowledge, develop preferences, and actually make food choices, the need to create “junk-free environments” for children has gained increasing support from health professionals, consumer advocates, and concerned political circles [10]. Attempts to steer children's preferences and food choices in a healthier direction, however, have limited success in an “obesogenic environment” [11], one that promotes unhealthy foodstuffs and offers limited incentives for healthy, active lifestyles. 

Although this “infrastructure of obesity” comprises many levels, is highly complex, and includes many interacting factors (see Butland et al.'s [12] influential 2007 Foresight report on tackling obesity in Britain), the key influential factors at work for children might, from a human ecological perspective, be roughly grouped by environmental type. Such ecological models, which consider individual behaviour in the context of multiple environments, offer a promising approach to obesity prevention [13–16]. In this paper, we focus on variables from the following three types of environments.

- *Social Environment*. Children are embedded in families, neighbourhoods, peer groups, schools, and child care facilities in which others influence their food preferences and practices by transposing their social norms and attitudes, food likes and dislikes, and consumption practices and affect their food habits through exposure and learning processes. These social groups also act as “communication buffers” between the children and the advertising and media messages that group members filter and evaluate.
- *Physical Environment*. Children are directly exposed to a physical environment that offers or limits opportunities for physical activity (e.g., neighbourhood bikeability and walkability), access to healthful foods (e.g., accessibility and availability of healthy food in schools), and access to media (e.g., a TV in the child's own room). Such an environment thus provides both drivers and barriers for actors—from parents to community and school officials—to build “choice architectures” for more health-promoting environments.
- *Media Environment*. The media environment and in particular commercial communication (e.g., food advertising and all kinds of stealth marketing) have been shown to shape food-related knowledge, attitudes, preferences, and practices both directly and indirectly. On a political level, regulation and self-regulation of advertising towards children are instruments that actively shape the media environment and potentially limit its influence on children's food preferences. A key moderating variable is children's advertising literacy or “ad smartness”, which increases with cognitive development and hence children's age.

This present study, although it acknowledges the multitude of influential factors and the interactions within these three environments, focuses on only a few key factors, whose selection was driven by one widely accepted and empirically based assumption: children's exposure to highly sophisticated advertising messages, including less blunt forms of subtle “stealth” marketing techniques, together with ubiquitous food availability that encourages the consumption of calorie-dense food products of low nutritional value, is a major cause of children's unhealthy dietary choices [17, 18]. The question, therefore, is not *whether *food marketing to children works, but *how* it affects them. A better understanding of this process is a precondition for developing effective consumer policy tools to protect children from overexposure and imprinting. To enhance such understanding, this paper analyses the associations between TV food advertising and children's food knowledge, food preferences, diets, and weight status. Specifically, it draws on data for a subsample of the IDEFICS study [19], 229 elementary school children aged between 6 to 9 years from five European countries. 

Before outlining our research design, we briefly sketch the key results of prior research on the impact of TV advertising on children's food knowledge and food preferences. Because the recent scientific literature offers comprehensive overviews on the state of the art in this field (e.g., [20–23]), we focus on the key variables in our study and their reported interrelations with each other and with advertising. Against this background, we develop our theoretical model and formulate the research hypotheses that guide our empirical study. After describing our methodology and analyses, we discuss our results as they relate to our hypotheses and conclude by outlining the policy implications of our findings. 

### 1.2. The Impact of Food Advertising on Food Knowledge and Preferences

Children in Europe and the USA are heavily exposed to mass media, watching over two and a half hours of television daily on average (e.g., [24]). Depending on the children's age and taking into account multiuse of media, recent reports show an average media exposition of 8-to-18-year olds in the USA of more than seven hours per day [25]. Because ad-free noncommercial children's TV channels like those in Germany and Sweden are the exception, these hours of viewing bombard children with advertising [26]. As a result, in the USA, foods consumed in front of the TV account for about 20–25% of children's daily energy intake [27]. In the EU, the Audiovisual Media Directive limits product placement and commercial sponsoring during children's programmes while still leaving member states adequate leeway in audiovisual media regulation; nevertheless, limits are stricter in some EU countries than in others [28]. No such regulation exists in the USA, however, where children aged between 2 and 11 are exposed to about 25,000 commercials per year, some during adult programming like soap operas or cooking shows [29]. In the USA, 20% of these commercials are for food products, 98% of them high in sugar, fat, and/or sodium [20, 28]. The same holds true for Europe where the “big five”—sugared breakfast cereals, soft drinks, confectionary, savoury snacks, and fast food outlets—represent the majority of advertised food [22]. There is ample empirical evidence that such advertising content often leads to unhealthier food choices [30]. In fact, research identifies a direct causal effect of exposure to food advertising on *children's diet*; in particular, an increase in snack [31] and overall calorie consumption [17], an immediately lower intake of fruits and vegetables [32], and higher rates of obesity [33].

There is also empirical evidence that food advertising affects *knowledge* about (un)healthy nutrition: commercials for unhealthy foods relate directly to lower levels of nutritional knowledge (e.g., [34]). Advertising, therefore, seemingly overrides knowledge already acquired from other sources that promote healthier choices. In fact, effective advertising messages, rather than requiring active processing and understanding, imprint positive associations on children's brains that can be triggered in decision situations [35]. Nevertheless, evaluations based on comprehensive literature reviews [22, 36] conclude that the overall direct effect of advertising on children's food knowledge and preferences is modest rather than strong. 

Empirical consumer research also shows that consumer knowledge does not necessarily lead to *preferences* for healthier food and that even if such preferences develop, they do not automatically guide behaviour. Thus, although most children and their families generally know what a healthy diet involves, their food choices often do not mirror this knowledge [37]. In fact, research indicates that accurate beliefs about food healthfulness are not associated with either food preferences or food consumption in children [38]. There is also evidence that the food choices of both children and their families are determined far more by attitudes and preferences than by acquired knowledge and that children are highly susceptible to the influence of peers in other social contexts [39]. Yet despite such evidence, prevention and intervention programmes usually take the educational approach [40].

Children's food preferences are also influenced by their immediate environment, particularly exposure to and familiarity with food stuffs, and by role models. Yet, according to the empirical literature [41], food advertising can influence children's preferences either way—healthier or unhealthier preferences [42]. Children also imitate their parents' (and other adult caretakers') food styles and learn by observation, meaning that they prefer eating fruits and vegetables if their parents do so. Their food preferences can also be influenced by sheer exposure to specific foods (the “I like what I know” phenomenon) [43].

## 2. The Study

### 2.1. The Human Ecological Model and Key Variables

**Figure 1:**
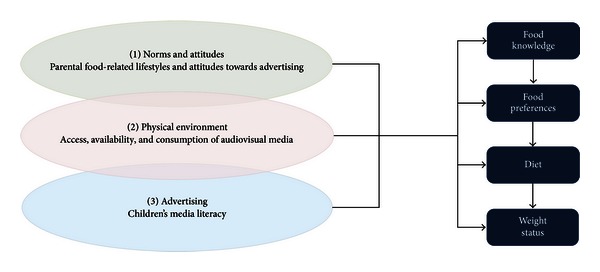
The role of commercial communication and food knowledge, preferences, diet, and weight status.

This study investigates the association between food advertising and children's food knowledge, food preferences, diet, and weight status, as summarised in Figure 1. In line with the theory of human ecological development (“ecological model”, [44]) and based on the literature sketched above, we select as our key variables potentially influential factors from the children's social, physical, and media environment; namely, food-related norms, attitudes, and lifestyles at home; the children's access to TV and consumption of TV commercials; and the children's level of advertising literacy. We also examine the relation between food knowledge, preferences, diet, and weight status. 

### 2.2. Food-Related Norms and Attitudes

We measure the food and media setting at home by parents' general attitude towards advertising [45] and hypothesise that the more sceptical parents are about food advertising, the less susceptible their children are to the effects of advertising on food knowledge, preferences, diet and weight status:

H1: the more sceptical the parental attitude towards advertising, the better their children's food knowledge, the healthier their food preferences and diet, and the lower their weight status.

We also take into account the suggestion put forward in the consumer socialisation literature that if parents discuss and reflect on the aims of advertising with their children—for example, while watching TV together—they can help their offspring develop the “advertising literacy” [32] that is part of an effective advertising defence model:

H2: the fact that parents discuss the TV programmes/ads watched with their children influences these children's food knowledge, food preferences, diet, and consequently, weight status.

### 2.3. Access to and Consumption of Television Advertising

The potential impact of TV advertising is influenced by three variables: children's access to media, their penchants for TV programmes that carry more or less advertising, and their actual exposure. Unrestricted access increases hours of actual media exposure and influences the time of exposure to advertising, factors that are further augmented by children having a television in their own bedrooms [46]. The country of residence and the type of programme watched also influence exposure to advertising. Assuming the so-called “mere exposure effect”, therefore—that is, that mere (and also incidental) exposure to advertising affects children's food knowledge and preferences—and accepting that advertising has the power to shape preferences [41], food knowledge should be lower [34] and preferences should be unhealthier when exposure is high. Such high exposure has consequences for both diet and weight status:

H3: unrestricted access and thus more exposure to advertising leads to lower food knowledge, unhealthier preferences, diets, and an unhealthier weight status.

### 2.4. Advertising Literacy

Children's handling of advertising depends on their advertising literacy—their knowledge about the goals and mechanisms of advertising—as well as on their attitudes towards advertising. In this context, knowledge refers to children's perceptions, including suspiciousness, of advertising's credibility and usefulness, whereas attitudes reflect the entertainment value that the advertisements hold for children [47]. Hence, following Livingstone and Helsper [32], we propose the following hypothesis:

H4: children's advertising literacy is related to their food knowledge, preferences, diets, and weight status; hence, higher advertising literacy is associated with better food knowledge, healthier preferences and diets, and lower weight.

### 2.5. The Relation between Food Knowledge, Preferences, Diet, and Weight Status

This study assumes a sequential relation between food knowledge, preferences, diet, and consequently weight status; that is, better food knowledge leads to healthier food preferences, which in turn lead to healthier food choices that are mirrored in a healthy weight status. As regards the effect of food knowledge on preferences, there is empirical evidence that in children accurate beliefs about food healthiness are not associated with food preferences or consumption [38]. Obviously, in the light of this finding, the widely held assumption that increased knowledge of healthy nutrition leads to healthier choices is a “misperception” [31, p. 223]. We therefore offer an alternative hypothesis:

H5: better food knowledge does not necessarily imply healthier food preferences (a), food preferences have no direct effect on dietary choice (b), and the latter has no significant effect on weight status (c).

## 3. Data and Methodology

### 3.1. Sample and Survey

The data used for our analyses were obtained in the context of the IDEFICS study, a prospective cohort study that began with a baseline survey in 2007/2008 and continued with a follow-up survey two years later [19]. The total IDEFICS cohort consists of 16,225 children aged 2 to 10 years from eight European countries. One unique feature of this study is that it employs a large number of objective measurements and supplements the questionnaire data with a large amount of laboratory data. For example, in the IDEFICS baseline survey, run between September 2007 and May 2008, parents described their children's lifestyle, television consumption habits, diets, parental attitudes and sociodemographic circumstances in a detailed self-administered questionnaire. A thorough physical examination was also conducted on all children in the sample to determine their amount of body fat, weight, height, and other health indicators [19]. To gather more specific information on the children's food knowledge and preferences as well as on their advertising literacy, between April and June 2009, we developed instruments (choice experiments and a questionnaire) and collected additional data for a subsample in five countries. Only children that participated in the experiments and the questionnaire are included in the present analysis. The resulting sample size is 229 children aged between 6 and 9 years (average age = 7.83; standard deviation (SD = .77)), 122 (53.3%) of whom are female. The participants are distributed as follows across the five countries: Belgium, 60 (26.2%), Estonia, 25 (10.9%), Germany, 48 (21.0%), Italy, 47 (20.5%), and Spain, 49 (21.4%). 

### 3.2. Food Knowledge and Preferences: A Choice Experiment

The data on children's food knowledge and preferences were gathered via a choice experiment (see Gwozdz and Reisch [48]) based on Kopelman et al. [37] but adapted to our research question and settings. The primary stimuli were two brochures showing 10 matched pairs of food cards; one picturing relatively healthy food, the other relatively unhealthy food. As shown in Table 1, these matched pairs belong to the same respective food category (e.g., “juice”). (To minimise unintended influences, it would have been preferable to have the children choose between real food products instead of pictures; however, because the experiments were carried out in five different countries in which the same products were not available or known to the children, doing so was not an option. Rather, to ensure comparability between the countries, we chose the pictures as feasible alternative.) 

The two-step experimental procedure included a preference test and a knowledge test. In the preference test, the children were asked, “Which food or drinks do you like best?” They then drew a smile (for “true”) or a frown (for “false”) for each matched pair according to their (forced-choice) preference. The knowledge test proceeded in a similar way. Again, the children drew a smile or a frown for each matched pair in reaction to the following question: “What do you think: Which food or drink is the healthier one?” This order was chosen based on pretest results showing that conducting the preference test first would reduce framing effects. 

Based on the children's choice experiment scores (i.e., whether they chose healthier or unhealthier foods and drinks from the 10 matched pairs), we built one indicator for food knowledge and another for food preferences. Both indicators range between 0 (no healthy food chosen) and 10 (only healthy food chosen). We also created a dummy variable capturing high knowledge or healthy preferences whenever a score equalled 6 or above, i.e., 1 “score > 6” and 0 “score ≤ 6”) (see [37]). 

### 3.3. Measurement of Variables

#### 3.3.1. Children's Diet

Our first diet measure reflects children's diet quality—including meal frequency, diet composition and variety, fast food consumption, and snack and beverage consumption—as well as family control [49]. This first dependent variable is a continuous variable that describes diet quality based on the Youth Healthy Eating Index (YHEI) [50], which ranges from 0 to 80, with a higher score signalling a more healthful diet. The YHEI, which measures food consumption and food-related behavioural patterns, is based on food frequencies, which in the IDEFICS survey are collected using the Children's Eating Habits Questionnaire (CEHQ) [51]. This latter asks parents for information on their children's food consumption of 43 predefined food categories, excluding foods served at school. The YHEI scores, therefore, measure solely the healthfulness of the diet under parental control. We do, however, also include meal pattern information from the CEHQ, such as frequencies of fast food consumption, breakfast at home or in school, and family dinners. 

Based on these data, we are able to replicate 10 of the 13 original YHEI dimensions, which are listed below with nutritional values in brackets

*Food types*:

1. whole grains (sources of fibre, vitamins, and minerals),
2. vegetables (sources of vitamins and minerals),
3. fruits (sources of vitamins),
4. dairy (sources of calcium),
5. snack foods (unnecessary energy),
6. soda and drinks (unnecessary energy),
7. margarine and butter (sources of fat).

*Food behavioural patterns*:

(8) fried foods outside home (high energy intake),
(9) eat breakfast (indicator of healthful dietary patterns),
(10) dinner with the family (indicator of healthful dietary patterns).

The original version of the YHEI also includes the dimensions “meat ratio”, “multivitamin use,” and “visible animal fat”, but these factors are not covered in the IDEFICS data. We calculate the YHEI using the sum of all available subscores for the 10 dimensions, the criteria for which are adapted from Feskanich et al. [50]. 

The other two dietary measures mirror the relative intake of sugar and fat and thus also reflect diet quality. Specifically, we calculated the weekly consumption frequencies of each of 12 foods and beverages that are high in sugar content and 17 foods and beverages that are high in fat and divided the weekly sugar and consumption by the individual's total consumed food frequencies (see [52]).

#### 3.3.2. Children's Weight Status

The last set of dependent variables relate to the children's lagged weight status (in the follow-up survey, i.e., weight status two years after the baseline survey in 2007/2008). The IDEFICS data set provides several anthropometric measurements related to body composition, all measured by trained nurses based on the same standard operating procedures (SOPs) in all countries. Again, we used three different models to capture the weight status, each based on a different dependent variable.

*(i) Model 1*. We consider the body mass index (BMI = weight in kilograms by squared height in meters) as a continuous variable, calculated as usual as a *z*-score according to the growth charts from the Centers for Disease Control (CDC) [53]. 

*(ii) Model 2*. As a second anthropometric measure, we used the corresponding *z*-scores of waist circumference, based on the growth charts of the International Obesity Task Force (IOTF) [54]. 

*(iii) Model 3*. As a third measure, we used body fat mass, which must be calculated based on fat mass, as derived from Bammann et al.'s [55] “four component model”, together with hip circumference and triceps skinfold. 

#### 3.3.3. Parental Norms, Attitudes, and Food Practices

Norms and attitudes reflect the influence of the “setting”; that is, the general parental attitudes toward advertising [56] and whether parents discuss the TV programmes watched with their child. Hence, parental attitudes towards advertising measure the perceived usefulness and credibility of ads, as well as the expected effects of the ads on their children. Borrowing from Diehl and Daum's [56] Attitudes Toward TV Food Advertising Aimed at Children scale (AFAC), we ran a factor analysis for identifying dimensions of parental attitudes towards advertising. (We carried out a principal component analysis with varimax rotation resulting in two factors. The eigenvalue is 1.44, the Kaiser-Meyer-Olkin measure is .651, and all factor loadings are above .405. Cronbach's Alpha for Factor 1 is .737 (four items) and for Factor 2 is .510 (three items).) The result was two factors with the following statements for Factor 1: ad usefulness and credibility: (a) TV food advertising is a good source of information for children and parents, (b) TV food advertising assists parents in their efforts to feed their child a healthy and balanced diet, (c) a child clearly understands just how good the product presented in TV advertising is, and (d) TV food advertising informs children and parents about things they would otherwise never learn about. For the second factor on the effect of TV food ads, we include the following three statements: (a) TV food advertising causes children and their parents to spend their money on unnecessary and sometimes even harmful products, (b) TV food advertising is largely responsible for the weight problems and bad teeth of many children, and (c) TV food advertising can hardly have an influence on what children eat and drink (reversed). Reversed items were recoded before the average score was calculated for each of the two dimensions. The question on discussing TV content with children is phrased as follows: “When watching TV, do you discuss the programme/ads with your child?” The variable is coded as a dummy: 0 = “never or sometimes”; 1 = “often or always”.

#### 3.3.4. Exposure to Media and Advertising

Other information for the direct advertising context stems from the IDEFICS baseline survey and comprises data related to the children's TV viewing habits: whether a television is available in the children's bedroom (dummy) and their weekly TV viewing time. 

#### 3.3.5. Children's Advertising Knowledge and Attitudes

The questionnaire used to measure children's advertising knowledge and attitudes is based on an instrument developed and validated by Diehl [47], which covers three dimensions, each measured by three questionnaire items: credibility, children's perception of TV advertisements as a useful source of information; suspiciousness, their questioning of commercial messages; and entertainment, the fun factor of watching commercials. The first dimension, the credibility and usefulness of food advertising, assesses whether children perceive TV advertisement as a useful source of information about foods and drinks. The hypothesis underlying the second dimension, suspiciousness toward food advertisements, is that if children are suspicious of TV food advertising, they will know not to trust any advertising content and will thus question commercial messages. The assumption underpinning the third dimension, the entertainment factor of TV advertising, is that children who are more suspicious and have less trust in the credibility of TV advertisements experience them as less entertaining. This latter implies that once children understand the mechanisms underlying advertising, they no longer enjoy watching them as much as before. To these three dimensions, we add an additional dimension, social desirability, measured on a four-point scale from “disagree fully” (−2) to “disagree” (−1), “agree” (+1), and “agree fully” (+2). Taking into account our respondents' young ages, we present the answer categories as pictograms (“smileys”) instead of words, expressing the respective nuances of (dis)agreement with more or less happy faces. The suitability of this instrument was demonstrated in pretests [48].

#### 3.3.6. Control Variables

The controls encompass socioeconomic status, indicated by the maximum parental education level (ISCED levels 1–6), child's age, child's sex, and country dummies. Thus, all analyses have been adjusted for these variables where child's age is introduced by three dummy variables: age <8 years, =8 years, and >8 years, the latter acting as the reference category. Child's sex is also expressed in form of a dummy variable (0 is male; 1 is female). For the five countries, we created five dummies, with Belgium acting as the reference group. 

### 3.4. Statistical Analysis

To meet the study goals we use STATA/SE 11 software to carry out a set of ordinary least squares (OLS) or probit regressions in which food knowledge, preferences, diet, and weight status are the dependent variables. In a first step, we estimate the following regression model:

(1) $$\begin{array}{c} F=\beta_{0}+\beta_{1}\text{DA}+\beta_{2}\text{IA}+\beta_{3}C+\beta_{4}D+\varepsilon , \end{array}$$

where *F* is a vector for our measure for food knowledge or preferences and may have either continuous or discrete variables as defined above. DA is a vector of direct advertising context factors, IA is a vector of indirect advertising context factors, *C* is a vector of child and family characteristics, and *D* is a vector of country dummy variables (five countries, with Belgium as the reference country). *ε* is a vector of idiosyncratic error terms, and the *β*s are the coefficients to be estimated, with *β*_1_ and *β*_2_ being the coefficients of particular relevance in this study. Depending on the nature of *F*, we use either ordinary least squares or a probit model. Because we assume that knowledge is associated with preferences, we estimate the model on preferences a second time, now including food knowledge as an independent variable (see Figure 1). 

In the next step, we first exchange the dependent variables knowledge and preferences with diet and then, in a third step, with lagged weight status. We then repeat the analyses. For weight status, we include an additional control variable in the form of a dummy variable indicating whether a child stems from the control or the intervention region in order to consider any possible intervention effect. For each analysis, we estimate two models for each dependent variable.

## 4. Results

### 4.1. Descriptive Statistics

Among the 229 children that participated in the choice experiment, the average score for food knowledge is 7.76 (SD = 1.18), higher than the average score of 4.78 (SD = 2.08) for food preferences, both measured on the same scale. Although 95% of the 229 children scored 6 or higher in the food knowledge experiment, only 33% chose 6 or more healthy foods in the preference experiment. As regards diet, the average YHEI is 49.6 on a scale between 0 and 80, the relative sugar intake is 27.9%, and the relative fat intake is 26.5%. As Table 4 also shows, diet quality and intake varies by country: Estonian and Spanish children show a higher diet quality and less sugar intake than children from the other countries. The lowest fat intake is among children from Italy and Spain. Belgian children, whose diet is comparatively high in relative sugar and fat intake, are the thinnest in the sample, with the lowest BMI, waist circumference, and fat mass. An overview of descriptive statistics of all used variables can be found Table 4.

Given the previously discussed assumption of a relation between food knowledge and preferences, we expect that both variables will influence children's diets and that diet in turn will be associated with weight. We therefore ran a correlation analysis for these dependent variables; however, we did not find any statistically significant correlation between food knowledge and food preferences (*r* = .109, *P* = .101). The diet variables themselves (YHEI, proportional sugar, and fat intake) are statistically significantly correlated—a high proportion of sugar or fat in the diet is linked to an unhealthful diet (YHEI) and vice versa—and as might be expected the strongest correlations occur between the weight status variables. Yet the correlation analysis reveals no indication of links between food knowledge and preferences and diet and weight status. 

As regards the remaining variables, parents perceive advertising on average as having medium credibility (*M* = 1.86, SD = .69 on a scale from 0 to 4) and relate food advertising to negative effects on children's health (*M* = 2.74, SD = .61). In terms of access to media, analysed in terms of bedroom equipment and TV consumption time, about 38% of the children in the sample have a television in their bedroom; however, there are large differences between countries. The majority of children in Italy (82.6%) and Estonia (64.0%) have a television in their bedroom, followed by about a third of children in Germany (33.3%), but far fewer in Belgium (15.0%) and Spain (14.9%). The children spend an average of 1.32 hours per day watching TV. 

The assessment of advertising knowledge and attitude, measured on a scale from −6 to +6, indicates that on average children feel more suspicious (*M* = 1.73, SD = 2.96) about advertising than they think it is credible or useful (*M* = .86, SD = 3.25) and entertaining (*M* = .13, SD = 2.82). In our sample, the most suspicious are the Italian children (*M* = 3.87, SD = 2.20) while the least suspicious are the Spanish (*M* = .87, SD = 3.17) and Estonian children (*M* = .79, SD = 3.05). The Spanish children also perceive advertising as entertaining (*M* = .90, SD = 3.20) and believe in its credibility as a source of information (*M* = 1.15, SD = 3.28) more than any other national group except for Belgian children (*M* = 1.78, SD = 2.97). The Estonian children are the most critical: they are the least entertained by advertising (*M* = −1.88, SD = 2.83) and perceive food advertising as the least credible (*M* = −1.40, SD = 3.28). Overall, we observe a variation by country; however, because our small sample size precludes any analyses stratified by country, we must rather rely on the inclusion of country dummies as control variables.

### 4.2. Associations between Advertising and Food Knowledge and Preferences

In this section, we investigate the associations between the variables discussed above: parental norms and attitudes, access and exposure (as well as advertising knowledge), and food knowledge, preferences, diet, and weight status.  Table 2 presents the estimates of the food knowledge and preferences regressions, those for the continuous dependent variables in columns 1 and 3 and those for the dependent dummy variables on knowledge and preferences in column 2 and 5. The results reported in columns 4 and 6 are for the models that include food knowledge as an independent variable.

As the table shows, there is an apparent significant relation between parental norms and attitudes: if parental attitudes towards advertising are critical (i.e., if they believe that food advertising has a negative effect on children's dietary behaviour), children's food preferences are more healthful (e.g., *β* = .271, SD = .149, column 5). Other parental attitudes, however, do not appear to be statistically significant. Access to media, on the one hand, shows no statistically significant effects: it seemingly plays no role in either food knowledge or preferences. Media literacy and food knowledge, on the other hand, are related along the entertainment dimension: children who are entertained by ads show also less healthy food knowledge than others. In terms of advertising, the statistical significance of advertising's credibility on food preferences is especially noteworthy: we find a highly significant negative effect of advertising's credibility on food preferences, meaning that children who are less sceptical of advertising have less healthful food preferences. Not surprisingly, given the prior finding in the correlation analysis of no relation between food knowledge and preferences, the introduction of food knowledge into the preference models (columns 4 and 6) does not improve the models: there is no change in adjusted *R* ^2^ and food knowledge is not significantly associated with food preferences.

### 4.3. Associations between Advertising, Food-Related Lifestyles, and Diet

Table 3 shows the results for the role of commercial communication on diet. On the one hand, we find that diet quality (YHEI) and fat intake are associated with parental norms and attitudes when such attitudes are critical of advertising. That is, in direct contrast to our expectations, the more critical the parents, the less healthful a child's diet and the higher the proportional fat intake. One possible reason for this unexpected finding could be social desirability effect, although a reactionary effect of children to parents' effort to make them eat healthily could also be at work. Such speculation, however, cannot be tested using the available data. On the other hand, we find no direct evidence of an influence of TV consumption on diet, although children with equipment in their bedroom show a higher proportion of sugar intake in their diet (e.g., one TV increases the share of sugar by 3.35%). Advertising literacy, however, is statistically significant in two cases: the more children feel entertained by advertising, the more healthful their diet—which once again stands in contrast to our expectations. The positive association between a higher credibility and usefulness of advertising and the relative high sugar intake, however, is in line with our fourth hypothesis (H4). Our introduction of food knowledge and preferences into the models (columns 2, 4, and 6) does show they have a statistically significant effect on diet and the adjusted *R* ^2^ indicates an improvement. 

### 4.4. Associations between Advertising and Children's Weight Status

We also ran regression analyses for estimating the relationship between advertising and children's weight status. The dependent variables are BMI (CDC, *z*-score), waist circumference (Cole, *z*-score), and relative body fat (kg/m^2^). We find no association between weight status and either parental norms and attitudes or the physical environment. We do show that children who are suspicious of ads have a higher BMI (column 2); however, only when diet factors are included. In fact, diet seems to have an influence on weight status; rather, counterintuitively, the proportional sugar intake is statistically significantly in relation to the lagged weight status, indicating that the higher the share of sugar in a diet, the lower the weight status. Neither the diet quality nor the proportional fat intake are statistically significantly associated with lagged weight status. 

In sum, our findings are rather mixed. Although some factors of the attitudes and norms environment show effects in the predicted direction on the healthfulness of food preferences and diet (diet quality and proportional fat intake), we find no robust associations between the physical environment and food knowledge, preferences, diet, or weight status. If we substitute TV consumption with audiovisual media (AVM) consumption (TV plus computer, game console consumption time), however, there is a statistically positive association between AVM time and weight status. The media environment (i.e., media literacy), however, seems to have the hypothesised effects on food knowledge and preferences but not on diet and weight status. 

## 5. Discussion and Conclusions

This analysis, based on a subsample from the IDEFICS study, examines the effects of advertising on children's food knowledge and preferences, as well as on dietary choices and weight status. For the sake of focusing on the role of commercial communication, we do neglect possible impacts of genetic as well as lifestyle factors—which may indeed modify appetite, food intake, and preferences—in our analysis. Both types of factors and their influence have been studied within the IDEFICS study and will be published elsewhere. The key findings of our study are that better food knowledge is not seemingly linked to healthier food preferences and diet apparently has no significant effect on weight status. Although we acknowledge that the study is limited in sample size and operationalisation of the variables is based on our own reasoning and hence could be debated, these key findings do stand on robust empirical ground based on the analysis presented in this paper. 

We interpret our results in light of the frequent claims that effectively countering harmful food marketing practices requires child awareness and understanding, paired with the ability and motivation to resist [31]. Many empirical studies, as well as evaluations of health intervention programmes, have indeed shown that providing information and education alone—the major policy strategy of recent decades—fails to successfully decrease advertising's effects on children. One reason that advertising literacy alone does not seem to help is that this knowledge only guides behaviour when it is accessed and used at the same time as the advertising stimulus, something that marketers carefully avoid. In addition, different processes of persuasion operate at different age levels—that is, at different perceptual stages and levels of advertising literacy—which age-specific advertising takes into account [32]. Yet, although consumer policy efforts to strengthen children's ability to resist food industry lures have been part of many educational programmes on media literacy and consumer competence-building since the 1970s, no effective “food marketing defense model” [31] has been developed. The findings of this study provide further evidence that any such effort must go beyond informational approaches. 

Overall, our findings support the contention that traditional policy strategies, based primarily on informational and educational goals, are insufficient to decrease the effects of advertising on children. Hence, although food smartness and advertising literacy will remain unquestioned goals of young consumers' socialisation, they cannot be expected to adequately guide behaviour in a healthier direction [57]. A more promising policy approach might lie in the tools of behaviourally informed social regulation suggested in the behavioural economics literature on “nudging” [58]. From this perspective, parents and caretakers should be aware of their decisive role as “choice architects”; as artisans who guide their children's selections by regularly offering healthful and attractive food and limiting their exposure to television and other sedentary behaviours. Hence, the old WHO motto “making the healthy choice the easy choice” should be reassessed and taken more seriously by everyone responsible for children's diet. Above all, food choices are strongly affected by the “triple A” of food items—availability, affordability, and accessibility—particularly if paired with and supported by social norms [59]. For instance, customer's food choices can be strongly influenced by the mere promotion of healthful food choices in “smart canteens” that offer the healthier choice as the default option [60]. This influence is, of course, no news for marketing professionals, but the power of context and the limited cognitive involvement of consumers in habitual consumer behaviours have too long been neglected by policy makers and health professionals alike. These latter particularly must recognise that a junk-free, nonobesogenic environment may be a necessary condition for successfully reducing obesity rates. 

## Figures and Tables

**Table 1:** Matched pairs of food cards based in part on Kopelman et al. [37].

Number	“Relatively healthy”	“Relatively unhealthy”
1	Sugar-free cereals (all kinds of sugar-free cereals)	Sugared cereals (including chocolate or crunchy cereals)
2	Water (all kinds of drinking water)	Coke (all kinds of coke)
3	Pasta (all types of noodle with home-made tomato sauce)	Pot noodle (all types of instant pasta soups or prepared pasta)
4	Cereal bar (low in sugar)	Chocolate bar (whole milk chocolate)
5	Roast beef (all types of fatless roast)	Beef burger
6	Strawberry yoghurt (all types of fruit yogurt, curd cheese, or buttermilk)	Strawberry cake (all types of fruit cakes)
7	Whole meal bread (all types of whole meal bread)	White bread (all kinds, including ciabatta, baguette, white toast)
8	Orange juice (100%) (all 100% fruit juices)	Orange squash (all types of fruit squash)
9	Potato (boiled or baked)	French fries (also fried potatoes, hash browns)
10	Orange (all types of fruits)	Orange ice (all types of fruit ice popsicles/lollipops)

**Table 2:** Role of commercials on food knowledge and preferences: OLS/probit estimates.

	Knowledge (1–10) OLS	Knowledge (>6, dummy) Probit	Preferences (1–10) OLS		Preferences (>6, dummy) Probit	
	(1)	(2)	(3)	(4)	(5)	(6)
(A) Parental norms and attitudes						

H1: attitudes towards ads (parents): usefulness and credibility	.071	.036	.014	.008	.089	.088
	(.123)	(.291)	(.243)	(.242)	(.148)	(.148)
H1: attitudes towards ads (parents): effects of ads	−.112	−.160	.266	.276	.271*	.272*
	(.106)	(.224)	(.254)	(.255)	(.149)	(.148)
H2: discussing TV programs with child	−.181	.017	−.211	−.195	−.107	−.108
	(.191)	(.433)	(.349)	(.351)	(.228)	(.229)

(B) Physical environment						

H3: TV consumption (hours per day)	.061	.268	.145	.140	.049	.048
	(.116)	(.220)	(.191)	(.193)	(.130)	(.130)
H3: bedroom equipment	−.286	−.457	.301	.327	.120	.206
	(.185)	(.386)	(.432)	(.440)	(.242)	(.243)

(C) Advertising						

H4: credibility dimension	−.031	−.031	−.034	−.032	−.056*	−.055*
	(.021)	(.041)	(.051)	(.052)	(.033)	(.033)
H4: suspiciousness dimension	.018	−.054	.027	.025	−.016	−.015
	(.024)	(.056)	(.059)	(.059)	(.036)	(.036)
H4: entertainment dimension	−.087***	−.097*	−.049	−.041	−.022	−.021
	(.027)	(.051)	(.059)	(.063)	(.038)	(.038)

(D) Food knowledge						

H5: food knowledge				.090		.191
				(.159)		(.468)

Observations	204	204	204	204	204	204
*R* ^2^	.276	.184	.080	.081	.069	.069
*F*-value/Wald *χ* ^2^	7.73	28.52	1.10	1.13	17.02	17.68
*P* value	.000	.008	.360	.334	.255	.280

Robust standard errors in parentheses; control variables are sex and age of child, parental education (ISCED), and country dummies. Reference category for age is 9 years and for countries, Belgium.

*P < .1; **P < .05; ***P < .01.

OLS: ordinary least squares estimator.

**Table 3:** Role of commercials on diet: OLS.

	YHEI		Relative sugar intake		Relative fat intake	
	(1)	(2)	(3)	(4)	(5)	(6)
(A) Parental norms and attitudes						

H1: attitudes towards ads (parents): usefulness and credibility	.177	.228	1.355	1.214	−.904	−1.584
	(.800)	(.869)	(1.190)	(1.320)	(.960)	(1.040)
H1: attitudes towards ads (parents): effects of ads	−.966	−1.298*	.941	.523	3.388*	2.682*
	(.674)	(.778)	(1.140)	(1.370)	(1.040)	(1.190)
H2: discussing TV programmes with child	1.270	1.443	−.565	.725	−2.800*	−2.640
	(1.190)	(1.390)	(1.600)	(1.950)	(1.380)	(1.620)

(B) Physical environment						

H3: TV consumption (hours per day)	−.843	−.941	.792	.809	.334	1.034
	(.658)	(.748)	(1.030)	(1.160)	(.908)	(1.010)
H3: bedroom equipment	−.341	−.207	3.354*	3.106	−1.505	−.922
	(1.190)	(1.350)	(1.790)	(1.970)	(1.540)	(1.760)

(C) Advertising						

H4: credibility dimension	−.234	−.301*	.735***	.534**	.343	.264
	(.144)	(.154)	(.216)	(.246)	(.210)	(.234)
H4: suspiciousness dimension	.100	−.013	.079	.272	.031	.303
	(.170)	(.197)	(.240)	(.272)	(.214)	(.238)
H4: entertainment dimension	.413***	.397***	.097	.031	−.124	−.224
	(.159)	(.192)	(.295)	(.345)	(.241)	(.281)

(D) food knowledge and preferences						

H5: food knowledge		.290		−.346		.478
		(.590)		(.895)		(.701)
H5: food preference		−.393		.301		.202
		(.239)		(.351)		(.295)

Observations	216	183	235	200	235	200
*R* ^2^	.147	.178	.143	.152	.143	.166
*F*-value/Wald *χ* ^2^	3.24	2.78	2.93	2.78	2.22	2.03
*P* value	.000	.000	.000	.000	.008	.013

Robust standard errors in parentheses; control variables are sex and age of child, parental education (ISCED), and country dummies. Reference category for age is 9 years and for countries Belgium.

*P < .1; **P < .05; ***P < .01.

OLS: ordinary least squares estimator.

**Table 4:** Descriptive statistics.

Variable	Obs.	Mean	Std. Dev.	Min.	Max.
Dependent variables					

Food knowledge	229	7.76	1.18	3	10
Food knowledge > 6	229	.95	.22	0	1
Food preferences	229	4.78	2.08	0	10
Food preferences > 6	229	.33	.47	0	1
Diet quality: YHEI	201	49.60	7.63	25.9	69.0
Relative sugar intake (%)	224	27.94	11.13	2.8	55.8
Relative fat intake (%)	224	26.52	1.00	6.1	58.7
BMI (CDC, *z*-score)	181	.25	1.12	−2.8	2.4
Waist circumference (Cole, *z*-score)	181	.68	1.19	−1.99	3.34
Relative body fat (kg/m^2^)	179	3.13	2.18	0	10.36

(a) Parental norms and attitudes					

H1: attitudes towards ads (parents): usefulness and credibility	225	3.14	.69	1	4
H1: attitudes towards ads (parents): effects of ads	225	2.31	.64	1	4
H2: discussing TV programmes with child	222	.35	.48	0	1

(b) Physical environment					

H3: TV consumption (hours per day)	224	1.32	.76	.07	4
H3: bedroom equipment	226	.38	.49	0	1

(c) Advertising					

H4: credibility dimension	227	.86	3.25	−6	6
H4: suspiciousness dimension	222	1.73	2.96	−6	6
H4: entertainment dimension	225	.13	2.82	−6	6

Controls					

Belgium	229	.26	.44	0	1
Estonia	229	.21	.40	0	1
Germany	229	.11	.31	0	1
Italy	229	.21	.41	0	1
Spain	229	.21	.41	0	1
Sex child	229	.53	.50	0	1
ISCED max.	228	3.72	1.11	0	6
