# Corrigendum to “Experimental Evidence on the Impact of Food Advertising on Children's Knowledge about and Preferences for Healthful Food”

**DOI:** 10.1155/2017/2826763

**Published:** 2017-07-12

**Authors:** Lucia A. Reisch, Wencke Gwozdz, Gianvincenzo Barba, Stefaan De Henauw, Natalia Lascorz, Iris Pigeot

**Affiliations:** ^1^Copenhagen Business School, Porcelaenshaven 18, 2000 Frederiksberg, Denmark; ^2^National Research Council, Institute of Food Sciences, Via Roma, 52 A/C, 83100 Avellino, Italy; ^3^Ghent University, De Pintelaan 185 Blok. A-2, 9000 Ghent, Belgium; ^4^University of Zaragoza, Domingo Miral s/n, 50009 Zaragoza, Spain; ^5^University of Bremen, Achterstraße 30, 28359 Bremen, Germany

In the article titled “Experimental Evidence on the Impact of Food Advertising on Children's Knowledge about and Preferences for Healthful Food” [1], there were errors in Section  1.2 titled “The Impact of Food Advertising on Food Knowledge and Preferences,” where the following corrections are applied.

(i) The following references should be added to the article with the numbers [61], [62], and [63]:

[61] L. M. Powell, R. M. Schermbeck, and F. J. Chaloupka, “Nutritional content of food and beverage products in television advertisements seen on children's programming,” Childhood Obesity, vol. 9, no. 6, pp. 524–531, 2013.

[62] J. Harris, J. A. Bargh, and K. D. Brownell, “Priming effects of television food advertising on eating behaviour,” Health Psychology, vol. 28, no. 4, pp. 404–413, 2009.

[63] K. A. Coon and K. L. Tucker, “Television and children's consumption patterns. A review of the literature,” Minerva Pediatrica, vol. 54, no. 5, pp. 423–436, 2002.

(ii) “As a result, in the USA, foods consumed in front of the TV account for about 20–25% of children's daily energy intake [27]” should be “In the USA, foods consumed in front of the TV account for about 20–25% of children's daily energy intake [27].”

(iii) Citation [28] in “In the USA, 20% of these commercials are for food products, 98% of them high in sugar, fat, and/or sodium [20, 28]” should be [61].

(iv) “In fact, research identifies a direct causal effect of exposure to food advertising on children's diet; in particular, an increase in snack [31] and overall calorie consumption [17], an immediately lower intake of fruits and vegetables [32], and higher rates of obesity [33]” should be “In fact, research identifies a growing evidence for a direct causal effect of exposure to food advertising on children's diet, in particular, an increase in snack consumption [31, 62]. Moreover, greater TV use—and therewith on average an increased food advertisement exposure—has been associated with an increase in overall calorie consumption [17], lower intakes of fruit and vegetables, and both the prevalence and incidence of obesity [63].”

(v) Citation [34] in “There is also empirical evidence that food advertising affects knowledge about (un)healthy nutrition: commercials for unhealthy foods relate directly to lower levels of nutritional knowledge (e.g., [34])” should be [38].

(vi) The format of reference [19] should be corrected as follows:

[19] W. Ahrens, K. Bammann, K. Buchecker et al., “The IDEFICS Study: design, participation, socio-demographic characteristics and compliance,” International Journal of Obesity, vol. 35, no. 1, pp. S3–S15, 2011.

Therefore, the section should read as follows:

Children in Europe and the USA are heavily exposed to mass media, watching over two and a half hours of television daily on average (e.g., [24]). Depending on the children's age and taking into account multiuse of media, recent reports show an average media exposition of 8- to 18-year-olds in the USA of more than seven hours per day [25]. Because ad-free noncommercial children's TV channels like those in Germany and Sweden are the exception, these hours of viewing bombard children with advertising [26]. In the USA, foods consumed in front of the TV account for about 20–25% of children's daily energy intake [27]. In the EU, the Audiovisual Media Directive limits product placement and commercial sponsoring during children's programmes while still leaving member states adequate leeway in audiovisual media regulation; nevertheless, limits are stricter in some EU countries than in others [28]. No such regulation exists in the USA, however, where children aged between 2 and 11 are exposed to about 25,000 commercials per year, some during adult programming like soap operas or cooking shows [29]. In the USA, 20% of these commercials are for food products, 98% of them high in sugar, fat, and/or sodium [20, 61]. The same holds true for Europe where the “big five”—sugared breakfast cereals, soft drinks, confectionary, savoury snacks, and fast food outlets—represent the majority of advertised food [22]. There is ample empirical evidence that such advertising content often leads to unhealthier food choices [30]. In fact, research identifies a growing evidence for a direct causal effect of exposure to food advertising on children's diet, in particular, an increase in snack consumption [31, 62]. Moreover, greater TV use—and therewith on average an increased food advertisement exposure—has been associated with an increase in overall calorie consumption [17], lower intakes of fruit and vegetables, and both the prevalence and incidence of obesity [63].

There is also empirical evidence that food advertising affects knowledge about (un)healthy nutrition: commercials for unhealthy foods relate directly to lower levels of nutritional knowledge (e.g., [38]). Advertising, therefore, seemingly overrides knowledge already acquired from other sources that promote healthier choices. In fact, effective advertising messages, rather than requiring active processing and understanding, imprint positive associations on children's brains that can be triggered in decision situations [35]. Nevertheless, evaluations based on comprehensive literature reviews [22, 36] conclude that the overall direct effect of advertising on children's food knowledge and preferences is modest rather than strong.

Empirical consumer research also shows that consumer knowledge does not necessarily lead to preferences for healthier food and that even if such preferences develop, they do not automatically guide behaviour. Thus, although most children and their families generally know what a healthy diet involves, their food choices often do not mirror this knowledge [37]. In fact, research indicates that accurate beliefs about food healthfulness are not associated with either food preferences or food consumption in children [38]. There is also evidence that the food choices of both children and their families are determined far more by attitudes and preferences than by acquired knowledge and that children are highly susceptible to the influence of peers in other social contexts [39]. Yet despite such evidence, prevention and intervention programmes usually take the educational approach [40].

Children's food preferences are also influenced by their immediate environment, particularly exposure to and familiarity with food stuffs, and by role models. Yet, according to the empirical literature [41], food advertising can influence children's preferences either way—healthier or unhealthier preferences [42]. Children also imitate their parents' (and other adult caretakers') food styles and learn by observation, meaning that they prefer eating fruits and vegetables if their parents do so. Their food preferences can also be influenced by sheer exposure to specific foods (the “I like what I know” phenomenon) [43].
